# Concept of Aided Phytostabilization of Contaminated Soils in Postindustrial Areas

**DOI:** 10.3390/ijerph15010024

**Published:** 2017-12-23

**Authors:** Maja Radziemska, Eugeniusz Koda, Ayla Bilgin, Mgdalena D. Vaverková

**Affiliations:** 1 Faculty of Civil and Environmental Engineering, Warsaw University of Life Sciences, Nowoursynowska 159, Warsaw 02-776, Poland; eugeniusz_koda@sggw.pl; 2 Faculty of Engineering, Artvin Coruh University, Seyitler Campus, Artvin 08000, Turkey; ayla.bilgin@artvin.edu.tr; 3 Faculty of AgriSciences, Mendel University in Brno, Zemědělská 1, Brno 613-00, Czech Republic; magda.vaverkova@uake.cz

**Keywords:** soil pollution, soil amendments, aided phytostabilization, chromium

## Abstract

The experiment was carried out in order to evaluate the effects of trace element immobilizing soil amendments, i.e., chalcedonite, dolomite, halloysite, and diatomite on the chemical characteristics of soil contaminated with Cr and the uptake of metals by plants. The study utilized analysis of variance (ANOVA), principal component analysis (PCA) and Factor Analysis (FA). The content of trace elements in plants, pseudo-total and extracted by 0.01 M CaCl_2_, were determined using the method of spectrophotometry. All of the investigated element contents in the tested parts of Indian mustard (*Brassica juncea* L.) differed significantly in the case of applying amendments to the soil, as well as Cr contamination. The greatest average above-ground biomass was observed when halloysite and dolomite were amended to the soil. Halloysite caused significant increases of Cr concentrations in the roots. The obtained values of bioconcentration and translocation factors observed for halloysite treatment indicate the effectiveness of using Indian mustard in phytostabilization techniques. The addition of diatomite significantly increased soil pH. Halloysite and chalcedonite were shown to be the most effective and decreased the average Cr, Cu and Zn contents in soil.

## 1. Introduction

The problem of contamination of the natural environment results largely from the enrichment of soils, waters and the air with trace elements (TEs), which are introduced into the environment as a result of anthropogenic activity—above all, the extraction of raw materials, the combustion of fossil fuels, treatment of sewage, storage of waste and industrial production [1,2]. Since soil components have a high capacity for retaining contaminants, the soil environment is one of the main recipients of TEs. In connection with the above, ecologically friendly techniques for the phytoremediation of TE-contaminated soil, among others using plants, have begun to appear and develop intensively over the last three decades [3]. Phytomanagement has been used more and more frequently as a technology for regaining heavy metals from soils. However, low solubility and creating permanent complexes in soil limit the phytoremediation of TEs from a contaminated environment [4,5].

**Figure 1:**
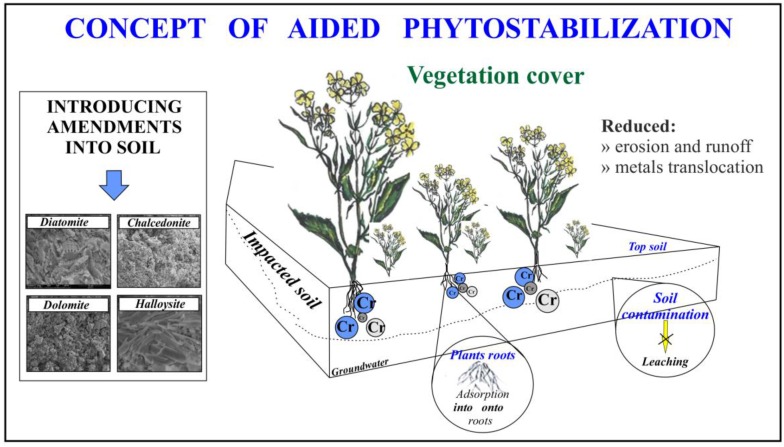
Concept of aided phytostabilization.

Applying phytomanagement in the form of phytostabilization is a suitable, economical and environmentally friendly technique, which facilitates the decreased threat to the natural environment connected with increased values of TEs [6]. Phytostabilization relies on, among others, permanent plant cover which may alleviate erosion and surface runoff [7], as well as minimizing the mobility and availability of TEs in the environment, preventing their transfer to subsequent links of the food chain and the migration of toxic ions to ground waters [8,9] (Figure 1). Phytostabilization is especially applicable in the case of metal contaminants at waste sites, where the best alternative is often to retain contaminants in one place. As opposed to other phytoremediation techniques, the aim of phytostabilization is not to remove heavy metals from the contaminated area but merely to stabilize them and reduce the risk to human health and the environment. The dominant mechanism thanks to which heavy metals are immobilized is the precipitation of hydroxides on a soil matrix. In this technique, additives (minerals/organic), which deactivate and, at the same time, immobilize TE ions in the substrate, are additionally applied [10]. The most widely applied amendments include clay minerals (i.e., sepiolite, palygorskite, bentonite), lime and limestone, phosphates, metal oxide, gravel sludge, red mud, zero valent iron, and organic composts [11,12,13,14].

Not many species of plants can be used for the phytostabilization of contaminated soils because soils in which increased concentrations of TEs occur may impair plant growth, leading to low crop yield, limited water retention abilities, etc. In connection with the above, native plant species are preferred for phytostabilization, as they are adapted to the contamination as well as local climatic conditions [15]. Plants used for phytostabilization are also characterized by low accumulation of TEs in the above-ground parts. Structural elements of the wall able to immobilize heavy metals, such as pectins, histidine groups and extracellular carbohydrates, are involved in preventing the transport of heavy metals across the cell membrane. The second strategy of protecting plants from the toxic effect of heavy metals is their intracellular detoxication. This relies on preventing the transport of metal ions across the cell membrane and their inactivation by binding in the cell and/or modifying them to less toxic forms [16].

Higher plants have the ability to absorb and neutralize chemically active contaminants found in soil. During their growth, they influence the concentration of heavy metals in contaminated soil in two ways, i.e., (1) by blocking the absorption of heavy metals by the roots to other plant tissues, and (2) by taking up, storing and immobilizing heavy metals through binding them with biologically active particles [17]. The choice of appropriate plant species for the needs of phytostabilization is the most important task preceding the remediation of a contaminated area. Indian mustard (*Brassica juncea* L.), which the authors used in the experiment, creates a taproot system, which penetrates deep into the ground, while its stalk is stiff. The flowers which it forms are bright yellow in color. Indian mustard grows best on soils which are abundant in lime, seeing as how its growth in acidic soil is poorer. Waterlogged and sandy soils are not suitable for its cultivation.

This experiment was performed to determine some of the parameters of the chemical composition of soil and plant growth under greenhouse conditions, and subsequently, to optimize soil conditions in order to improve plant growth for better success of the planned phytostabilization field measure. The novelty of this study is, on the one hand, the comparison of the application of novel trace element immobilizing soil amendments, and, on the other hand, the comprehensive analysis of the chemical composition of the plant-soil system. The objectives of the present study were (i) to observe changes in the physico-chemical properties of the tested soil in order to understand the translocation and accumulation of Cr in the soil and Indian mustard, and (ii) to compare the effectiveness of various soil additives on various chemical properties of the plant-soil system.

## 2. Materials and Methods

### 2.1. Contaminated Soils and Amendments

**Figure 2:**
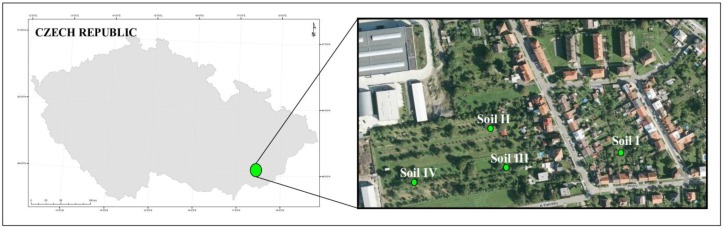
Sampling site location (**Soil I:** 49°09′41.9′′ N, 17°30′59.0′′ E; **Soil II:** 49°09′42.8′′ N, 17°30′50.3′′ E; **Soil III:** 49°09′40.9′′ N, 17°30′51.8′′ E; **Soil IV:** 49°09′40.7′′ N, 17°30′45.9′′ N).

The soil samples were taken from a post-industrial area, located in the Zlín Region of the south-east part of the Czech Republic. In the first half of the 19th century, the area experienced industrial development (sugar refinery, brickworks, brewery etc.). Since 1900, there has been a farm machinery manufacturing plant and engine manufacturing plant in the area. The distribution of soil pollution characteristics has been affected by past industrial activities. Nowadays, the study area in the impact zone has a new industry, mainly an aluminium smelter. The soil used in this study was sampled from the sub-soil found in the layer between 10 cm and 30 cm below the surface. All soil samples were randomly collected from sites in the post industrial area in May 2016 (Figure 2). At each sampling site, a composite soil sample (approximately 5.0–5.5 kg) was obtained by thoroughly mixing 4 subsamples. Stones, sticks and roots were manually removed. Soil samples were then stored in polyethylene bags and transported to the laboratory. The soil samples (I–IV) from the study area were contaminated with different levels of Cr. Table 1 shows the physico-chemical characteristics of soils used in the phytostabilization experiment.

**Figure 3:**
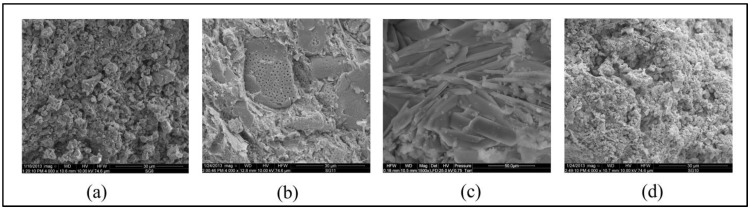
Scanning electron microscope (SEM) micrographs of dolomite (**a**), diatomite (**b**), halloysite (**c**), chalcedonite (**d**).

Scanning electron microscope (SEM) micrographs of dolomite, diatomite, halloysite, and chalcedonite are shown in Figure 3. Table 2 presents some properties of soil amendments.

### 2.2. Experimental Design

The pot experiments were implemented under greenhouse conditions (23 °C, humidity of approx. 60%). Polyethylene (PVC) pots were filled with 5 kg of air-dried soil and mixed with immobilizing soil amendments (dolomite, diatomite, halloysite, chalcedonite) 3% (*w*/*w*). The soil samples were thoroughly mixed and pots were placed in a dark room for over 2 weeks with 70% water holding capacity (WHC) to equilibrate the soil mixture. Soils without amendments (0.0%) were designated as the control. In previous studies [18], the addition of 3% additives was found to be the most effective treatment in immobilizing trace elements. Each treatment was replicated thrice. The experimental pots were arranged randomly in the greenhouse. Seeds of Indian mustard cv. Małopolska were obtained from an authorized Seed Production Centre in Olsztyn, Poland (OLZNAS-CN Sp. z o.o.), and five seeds planted per pot. The plants were watered every other day with demineralized water to 60% maximum water holding capacity of the soil. Indian mustard was harvested in the flowering phase. After 97 days, plants were harvested and separated into shoots and roots. The rhizosphere soil tightly adhering to the roots was collected by brushing off, air-dried and sifted through 2 mm sieves to determine Cr.

### 2.3. Soil Analytical Methods

The pH was determined in distilled water extracts (1:2.5 *w*/*v*) using a pH-meter (HI 221). Soil organic matter was determined by Tiurin’s method after hot digestion of soil samples with K_2_Cr_2_O_7_ and H_2_SO_4_ in the presence of Ag_2_SO_4_ as a catalyst and the titration of K_2_Cr_2_O_7_ excess with FeSO_4_/(NH_4_)2SO_4_·6H_2_O [19]. Upon completing the experiment, to determine trace element content in soil samples, 0.5 g of sieved soil were mineralized into a solution using a microwave oven (Milestone Start D, Bergamo, Italy) with a mixture of concentrated (69–71%) HNO_3_ and concentrated (37%) HCl (9:3 mL), in a partial digestion procedure equivalent to the USEPA Method 3051 A [20]. Certified reference material (Sigma Aldrich Chemie GmbH, No. SQC001, St. Louis, Missouri, MO, USA) was used for the analyses. The exchangeable soil metal fractions were determined using 0.01 M CaCl_2_ (1:10 soil-extractant ratio) after agitation for 2 h at 20 °C. The extract was separated from the solid residue by centrifugation for 15 min. [21].

### 2.4. Chemical Analysis of Plant Material

Before analysis, the plants were powdered using an analytical mill (Retsch type ZM300, Hann, Germany) and kept at ambient temperature in clean containers prior to chemical analyses. The roots and shoots were oven-dried at 55 °C to a stable weight, and the dry biomass was recorded. A representative subsample (0.200 g) was digested in nitric acid (HNO_3_ p.a. Chempur, Poland) with a concentration of 1.40 g·cm^−1^ and 30% H_2_O_2_ (Merck, Darmstadt, Germany) using a microwave oven (Milestone Start D, Bergamo, Italy). After filtration, the digestion products were adjusted to 100 mL volume with Milli-Q water (18.2 MΩ, Milli-Q Element A10 purification system, Millipore, Billerica, MA, USA). Extracts were analyzed for TE concentrations determined by the Atomic Absorption Spectrometry (AAS) method using an iCE-3000 spectrophotometer (Thermo Scientific, Waltham, MA, USA). Laboratory equipment was acid-washed (20% HNO_3_) and rinsed with deionized water. All reagents were of analytical reagent grade unless otherwise stated. Each sample was processed in triplicate.

### 2.5. Data Analysis

The accumulation of TEs in Indian mustard can be assessed by two factors: (1) the bioconcentration factor (BCF) and (2) the translocation factor (TF). The BCF is calculated respectively as the ratio of trace element concentration in the roots of plants at harvest to the concentration of the element in the soil [22]. The TF is calculated as the ratio of the element concentration in the shoots to the roots, and indicates the ability of a plant to translocate metals upward from its roots to its shoots [23]. Values of TF > 1 represent the effective translocation of metals from the roots to the shoot [24].

Graphical analysis, analysis of variance (ANOVA), correlation analysis, and multivariate analysis {Principal Component Analysis (PCA)} of data were carried out by SPSS-20 (SPSS Inc., Chicago, IL, USA) and XLStat (Addinsoft, Paris, France).

## 3. Results

### 3.1. Plant Growth

**Figure 4:**
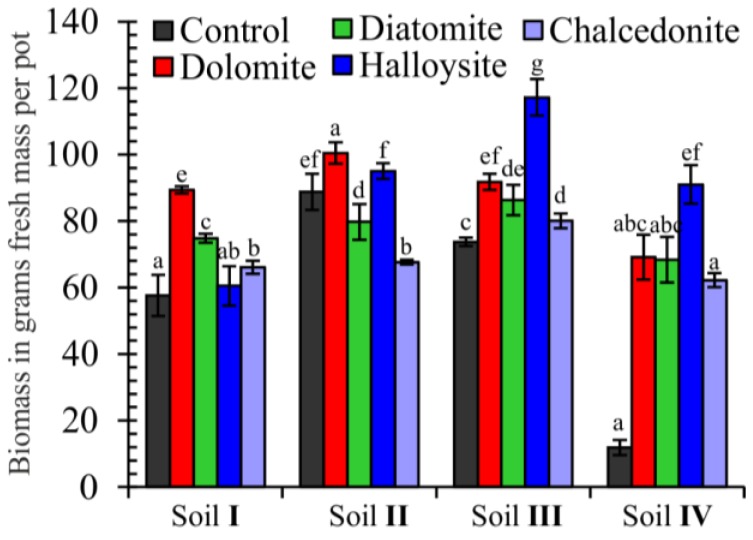
Effect of Cr contamination, and soil amendments on the above-ground biomass of Indian mustard in grams of fresh mass per pot. Error bars are ± standard error (*n* = 3). Bars marked with different letters differ significantly for the same heavy metals exposure (*p* < 0.05) according to the Duncan test.

As shown in Figure 4, the above-ground parts of the tested plant in the control series (without amendments) grown in the soil with the highest level of Cr (Soil IV) were characterized by high sensitivity to soil contamination, which is confirmed by the occurrence of a negative correlation between plant yield and increasing soil contamination with Cr. The soil with the highest level of Cr caused a decrease in the mass of aerial-parts to 80% as compared to the control. The greatest average above-ground biomass was observed in cases of amending soil with halloysite (51%) and dolomite (46%) as compared to not applying any of these substances to the soil. Diatomite and chalcedonite also had a positive, though lesser, influence.

### 3.2. Cr Accumulation and Translocation

**Figure 5:**
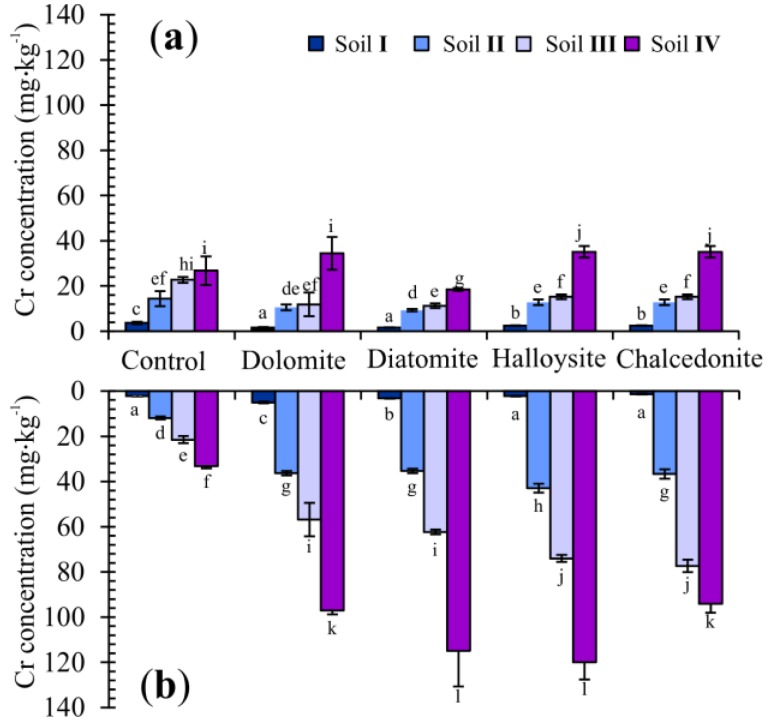
Chromium concentration (mg·kg^−1^, dry weight basis) in the above-ground part (**a**) and roots (**b**) of Indian mustard at the end of the trial. Error bars are ± standard error (*n* = 3). Bars marked with different letters differ significantly for the same Cr exposure (*p* < 0.05) according to the Duncan test.

In the present study, the concentration of Cr in the roots and above-ground parts of Indian mustard was closely correlated with the level of Cr and amendments (dolomite, diatomite, halloysite, and chalcedonite) introduced into the soil (Figure 5). In the control, Cr concentrations (in mg·kg^−1^ dry weight) varied between 3.67 to 26.77 in the shoots, and between 2.22 to 33.21 in the roots. Compared with the control treatment, the halloysite and diatomite treatments significantly increased Cr concentration in plant roots as compared to pots to which additives had not been added.

**Figure 6:**
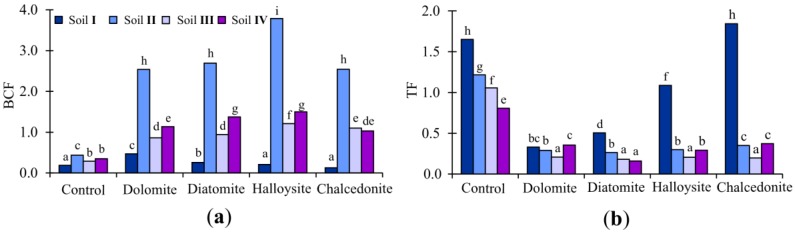
Effects of applying soil amendments on Cr accumulation (BCF) (**a**) and translocation (TF) (**b**) in Indian mustard.

The results obtained for BCF and TF are reported in Figure 6. Our findings suggest that, based on the values of two important major factors, i.e., BCF and TF, the proposed set of mineral additives and plants (Indian mustard) form a system suitable for Cr phytostabilization. BCF values of Indian mustard ranged from 0.21 to 3.79 in halloysite treatment, while TF values ranged from 0.16 to 0.50, and were the lowest in the case of diatomite treatment. 

### 3.3. Effects of Amendments on Chemical Properties of Soil

**Figure 7:**
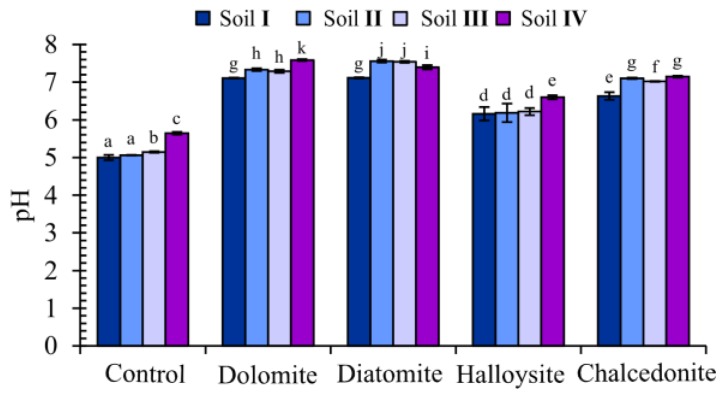
Results of soil pH obtained for the tested amendments; (mean ± SD, *n* = 3). Values in columns marked with the same letter do not differ significantly (Dunkan test, *p* > 0.05).

In the presented research, the pH of soil solutions increased following the addition of amendments (Figure 7). The greatest increase was of 2.19 pH units, observed after diatomite had been added to the soil.

**Figure 8:**
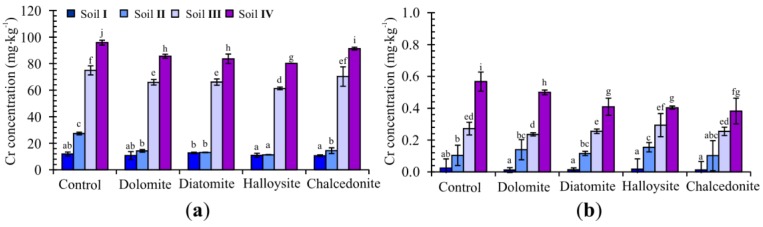
Total (**a**) and CaCl_2_-extractable (**b**) Cr fractions in soil with different soil treatments (mean ± SD, *n* = 3). Values in columns marked with the same letter do not differ significantly (Duncan test, *p* > 0.05).

Soil total and CaCl_2_-extractable Cr concentrations have been presented in Figure 7. In this study, the application of halloysite and diatomite led to a significant decrease in total Cr concentrations in soil as compared to the control pots. The concentration of CaCl_2_-extractable Cr in treatments with amendments was significantly lower than total content (Figure 8). Applying immobilizing amendments to the soil contributed to decreased levels of Cu, Zn, and Ni total concentrations as compared to the control series, i.e., without additives (Table 3). The application of amendments reduced CaCl_2_-extractable content of TEs in soil, with the most significant reduction observed for chalcedonite treatment. Compared with the control, treatment with this amendment reduced CaCl_2_ adding halloysite, although its influence was weaker.

### 3.4. Pearson’s Correlations

The correlation matrix between the physico-chemical properties of soil- and plant-related parameters, calculated using data obtained from PCA, is shown in Table 4. Accordingly, there is a significant and positive relationship between Cr concentration in the above-ground parts and roots of Indian mustard, and total Cr, Cu and CaCl_2_-extractable Cr, Cu (*r* = 0.519 to 0.757) in the tested soil. Moreover, a significant and positive relationship between soil pH and CaCl_2_-extractable Zn (*r* = 0.501) was observed. There is a significant and positive relationship between the total content of Cu and Cr in above-ground parts of tested plants, and the total and CaCl_2_-extractable Cr and Cu in soil (*r* = 0.546 to 0.620). There is a significant and positive relationship between CaCl_2_-extractable Cu and Cr, and Cr in above-ground parts of Indian mustard (*r* = 0.519 to 0.673).

### 3.5. Statistical Analysis

According to the ANOVA analysis, carried out to identify differences in the data, the soil parameters, i.e., pH, CaCl_2_-extractable Cu, and Zn concentrations, as well as pseudo-total contents of Zn and Ni were *p* < 0.05, indicating a statistically significant difference (Table 5). The *p* < 0.05 conclusion at the end of the ANOVA analysis indicates a significant difference for four parameters. The ANOVA analysis shows that there is a significant difference between the control pots and pots to which amendments (dolomite, diatomite, chalcedonite) had been applied when it comes to the pH parameter of soil. Significant differences were indicated between total Cu in the control soil and soil from pots containing chalcedonite. Such differences were also indicated between the control soil and soil from pots containing dolomite and halloysite in regards to Ni in soil, and dolomite in regards to CaCl_2_-extractable Zn.

**Figure 9:**
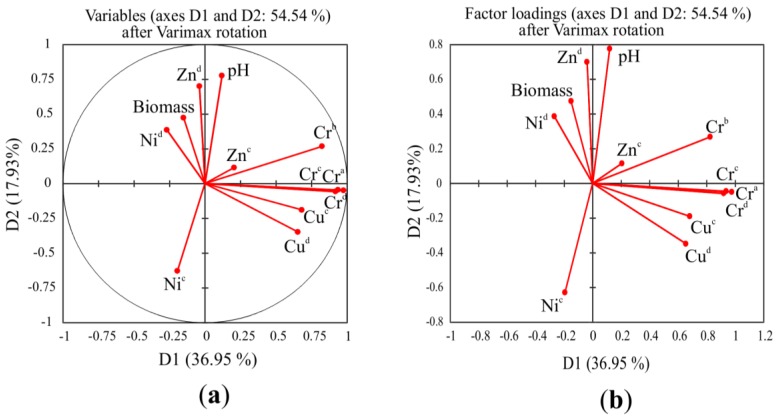
Plot of scores of principal component. (**a**) Variable after varimax rotation. (**b**) Factor loading after varimax rotation. ^a^ metal concentrations in the above-ground plant material. ^b^ metal concentrations in the root; ^c^ total content in soil; ^d^ CaCl_2_-extractable metal concentration.

Suitability of data for factor analysis is tested using the Kaiser-Meyer-Olkin (KMO) and Barlett Tests. According to the results of the analysis, a value of KMO = 0.5 is suitable for use in this analysis. According to factor analysis, four factors were identified with eigen values greater than 1, the ratio to total variance of which showed a gradually decreasing tendency (Figure 9). These four factors explain 76.76% of the total variance. The first factor (F1) explains 37.16% of total variance, and Cr concentrations in above-ground parts and roots of Indian mustard, total content of Cr in soil, and CaCl_2_-extractable Cr concentration having a strong positive load, while total Cu concentration in soil and CaCl_2_-extractable Cu having moderate positive load values. The second factor (F2) explains 17.7% of total variance, with soil pH, CaCl_2_-extractable Zn in soil, and the biomass of the tested plant exhibiting strong positive, moderate positive, and moderate negative load values, respectively. The third factor (F3) explains 12.43% of total variance; total Zn concentration in the soil has a strong positive load value. Finally, the fourth factor (F4) explains 9.79% of total variance, with total Ni content found to have a moderate positive load value.

## 4. Discussion

The proper development of plant habitats in areas contaminated by TEs is greatly dependent on the physicochemical properties of soil. The condition that must be met in order for contaminants to be immobilized using the technique of aided phytostabilization is creating adequate vegetation cover on the contaminated land [25]. In the case of Cr, both its shortage as well as excess in soil can lead to negative effects in plants; e.g., its shortage can be the cause of, above all, the inhibition of growth and photosynthesis, as well as decreased resistance of the plant to pathogens, whereas when added to soil, it can improve plant yield [26]. In the present research, soil with the highest level of Cr caused a decrease in the mass of above-ground plan parts to 80% as compared to the control. This is confirmed by earlier studies by Wyszkowski and Radziemska [27], in which Cr(VI) led to a reduction in the dry mass of plants. Similarly, Shankera et al. [28] and Golovatyj et al. [29] reported that the production of maize and barley decreased due to the presence of Cr. Positive effects of applying mineral immobilizing additives to soil contaminated with TEs are also confirmed by other authors, i.e.: Molla et al. [30], Radziemska et al. [31,32], Sun et al. [33], and Szostek and Ciećko [34].

As reported by Μolla et al. [30], and Wyszkowski and Radziemska [35], Cr content in plants is connected with a set of chemical and geological factors, though the content of chromium compounds in soil plays an important role. This means that the content of Cr in plants is dependent on both, the abundance of this element in soil, as well as the plant species [27]. Furthermore, the phytotoxic effects of Cr are primarily dependent on the speciation of the metal, which determines its uptake, accumulation and translocation [36]. Dheri et al. [37] found that fenugreek, spinach and field mustard achieved respective concentrations of 3.4, 5.5, and 8.2 mgCr·kg^−1^ per 10 mg·kg^−1^ of added Cr to soil. Kanwar et al. [38], on the other hand, found the concentration of Cr to be 36–42 mg·kg^−1^ in Indian mustard plants. In our studies, the accumulation of Cr was much higher in roots than in shoots. The taken up TEs accumulate in greater amounts in the roots than in the shoots, proportionately to the increase of concentration in the soil, with up to 70–98% of the taken up metals remaining in the roots [39]. The uptake of metals from the soil into the roots is dependent on the bioavailability of the metal, as well as its mobility in the rhizosphere [40]. This relationship is also confirmed by studies carried out by Μolla et al. [30], where zeolite and bentonite additions to soil as amendments reduced total Cr levels in Zea mays compared to the control. In another experiment conducted by Radziemska et al. [18], limestone and chalcedonite caused significant increases of TE concentrations in the roots of rye grass. Feng et al. [41] demonstrated that soil amended with minerals, i.e., bentonite, clays and zeolite, effectively restrained the impact of Zn on Yellow lupine. Indexes of the bioconcentration (BCF) and translocation factor (TF) are a useful tool for interpreting the accumulation and mobilization of metals in plants. A BCF higher than 1 indicates that the element considered tends to accumulate in the roots of the plant [42], whereas plants with low TF values are considered to be useful for phytostabilization [43]. The translocation factor values for ryegrass varied minimally, i.e., 0.06–0.07, in an experiment by Abad-Valleet al. [44] in which sepiolite was added to soil contaminated with heavy metals. In a different experiment by Radziemska et al. [18], BCF values for the test plant ranged from 0.93 to 1.73 in chalcedonite treatment, whereas TF values were between 0.05 and 0.35, and were the lowest in the case of limestone treatment.

The mobility of TEs in soil pertains to, above all, absorbable and bioavailable forms for plant organisms, which may permeate to the soil solution. The mobility of TEs in the soil environment is determined by the forms of their occurrence as well as the mechanisms by which they bind with organic as well as inorganic soil components. In order to limit the mobility of heavy metals and to ensure stabilization in the soil environment, substances of an organic and/or mineral character are introduced into the soil. The solubility of all metals is strongly dependent on the organic matter of soil, cation and anion exchange capacities, texture (clay content), soil type, redox potential and pH value of soils [45]. The solubility of the majority of metals decreases along with increasing pH, which in turn leads to lowering their bioavailability. In acidic soils, an increase in the availability of mobile forms of heavy metals in the soil solution occurs [46]. This is connected with an increase in the solubility of chemical bonds of these elements as well as their decreased absorption on soil colloids at low soil pH [47]. In the present research, the greatest increase was observed after diatomite had been added to the soil. This is in line with the results of Ye et al. [48] and Radziemska et al. [18], who found that the addition of natural and modified diatomite increased the pH value of soil. The mobility of heavy metals in the soil environment is determined by the form in which they occur as well as the mechanisms of their bonds with organic and non-organic soil components. The conditions occurring in the soil are also very important, especially physical as well as chemical properties, which significantly influence the mobility of trace elements and their assimilability by plants [32]. The mechanisms by which the amendments immobilize metals are based on sorption to particles, alkaline properties, and co-precipitation reactions [49]. The concentration of CaCl_2_-extractable Cr in treatments with amendments was significantly lower than total content. This suggests that soils treated with the application of the tested amendments exhibit a greater ability to desorb Cr from the soil in comparison to soil without additives. The amendments applied in the experiment immobilize metals via some mechanisms such as: adsorption of heavy metals to highly accessible sites on the surface of amendments and the components of the soil, and due to the amendment liming effect. If soil amendments are used to enhance immobilization, they may need to be periodically reapplied to maintain their effectiveness [50]. Thanks to the immobilization of TEs in soil, it is possible to return physicochemical as well as functional properties to degraded land. The land is no longer able to be used for food production. In contrast, some engineering technologies, such as soil replacement, can return the site to its former land use [51]. In another experiment conducted by Radziemska and Mazur [31], the addition of halloysite to soil contaminated with TEs caused a significant decrease of Ni, Cr, Cu and Zn in the soil. A similar situation took place in the case of adding diatomite, chalcedonite and limestone to the soil, where the share of tested total and CaCl_2_-extractable TEs decreased significantly in an experiment by Radziemska et al. [18].

One of the major issues with phytostabilization technology is ensuring a successful operation that entails the contaminants remain on site. The main advantage of this technology is that it reduces their mobility and, therefore, the risk of contamination without necessarily removing them from their source location. Finally, to conclude, the present studies confirm the effectiveness of using novel immobilization materials, including dolomite, diatomite, halloysite and chalcedonite in the technique of aided phytostabilization of soil contaminated with Cr. When comparing information contained in the empirical part of the work as well as relaying the findings of various authors, it can be concluded that the studies carried out under conditions of a controlled pot experiment ought to be continued in field conditions.

## 5. Conclusions

The phytostabilization of contaminants is heading in the direction of creating a vegetation cover which protects the soil against erosion, migration of contaminants into the soil profile or on its surface along with the runoff of rainwater. In connection with the above, plants used in the method of phytostabilization should immobilize heavy metals in roots and not transport them to the above-ground parts due to the possibility of their further mobilization in the food chain. The present study clearly demonstrates novel evidence with respect to the aided phytostabilization of Cr-contaminated soils, demonstrating that the combined effect of Indian mustard and immobilizing amendment application may be a suitable approach to reducing environmental risk. The biomass of tested plants in particular organs depended on the level of the Cr contaminant and amendments incorporated into the soil. In this experiment, Cr accumulated predominantly in the roots of the tested plant. Halloysite and diatomite caused significant increases of Cr concentrations in the roots. The greatest average above-ground biomass was observed for soil amended with halloysite and dolomite.

Soil additives can be applied in an effort to accelerate the process of creating vegetation cover in soils where TE contamination is present, improve the quality of soil and the effectiveness of the immobilization of contaminants. In the experiment carried out by the authors, the application of immobilizing amendments tended to reduce the soil mobile fraction of trace elements (Cu, Zn, Ni) more in comparison to unamended soil. The present study clearly demonstrates that the combined effect of Indian mustard and halloysite application may be a suitable approach to the aided phytostabilization of Cr contaminated soils.

## Figures and Tables

**Table 1:** Selected parameters of the soil used in the phytostabilization experiment.

Parameter	Unit	Value			
		Soil I	Soil II	Soil III	Soil IV
pH	-	5.72	5.56	5.14	5.25
Organic matter	% d.m.	11.12	12.21	14.11	11.25
Chromium	Mg·kg^−1^ d.m.	10.14	48.22	99.24	148.21
Copper	mg·kg^−1^ d.m.	20.14	21.11	21.22	20.78
Nickel	mg·kg^−1^ d.m.	6.22	7.22	6.54	6.47
Zinc	mg·kg^−1^ d.m.	35.22	38.22	32.14	33.41

The mean values of three replicates are shown.

**Table 2:** Properties of soil amendments.

	Specific Surface Area (m^2^·g^−1^)	Chemical Composition in Oxide (wt. %)
Dolomite	1.22	CaO-38.12; N_2_O_2_-22.30; C_2_O-20.18; SiO_2_-6.91; Al_2_O_5_-4.51; Fe_2_O_3_-4.41; CuO-2.28%; MgO-1.29
Diatomite	29.3	SiO_2_-54.72; Fe_2_O_3_-25.50; Al_2_O_5_-14.82; C_2_O-4.18; MgO-0.79
Halloysite	49.5	SiO_2_-39.6; Al_2_O_3_-37.0; Fe_2_O_3_-16.1; TiO_2_-2.30; CaO-0.66; MgO-0.13; Na_2_O-0.04; K_2_O-0.05; P_2_O_5_-0.52
Chalcedonite	7.44	SiO_2_-84.77; Al_2_O_5_-9.33; C_2_O-4.29; K_2_O-1.21; MgO-0.40

**Table 3:** Total and CaCl_2_-extractable Cu, Zn, and Ni concentrations in soils subjected to different treatments (mean ± SD, *n* = 3).

Soil	Control	Dolomite	Diatomite	Halloysite	Chalcedonite
Total/CaCl_2_-extractable metal concentration					
Cu (mg·kg^−1^)					
Soil I	21.07 ± 0.78 0.26 ± 0.06	17.64 ± 0.28 0.20 ± 0.01	23.21 ± 0.95 0.17 ± 0.08	18.25 ± 0.13 0.18 ± 0.09	18.37 ± 0.67 0.17 ± 0.05
Soil II	22.48 ± 0.65 0.27 ± 0.05	18.92 ± 0.76 0.24 ± 0.08	22.57 ± 1.58 0.29 ± 0.22	20.97 ± 0.63 0.22 ± 0.10	18.83 ± 0.54 0.18 ± 0.01
Soil III	22.62 ± 0.15 0.35 ± 0.20	22.33 ± 1.12 0.26 ± 0.03	21.55 ± 1.75 0.29 ± 0.11	21.95 ± 0.55 0.28 ± 0.08	19.65 ± 0.57 0.18 ± 0.01
Soil IV	24.89 ± 0.08 0.41 ± 0.08	23.92 ± 0.53 0.31 ± 0.08	24.07 ± 0.35 0.26 ± 0.25	22.94 ± 1.47 0.30 ± 0.04	21.30 ± 1.04 0.19 ± 0.03
Zn (mg·kg^−1^)					
Soil I	39.35 ± 0.93 1.11 ± 0.02	38.43 ± 1.16 0.85 ± 0.02	32.38 ± 1.04 0.88 ± 0.01	33.30 ± 0.98 0.85 ± 0.03	31.72 ± 0.86 0.68 ± 0.03
Soil II	36.37 ± 1.07 0.96 ± 0.05	41.76 ± 1.88 0.78 ± 0.03	34.49 ± 1.12 0.96 ± 0.05	33.72 ± 0.65 0.88 ± 0.06	33.96 ± 1.48 0.88 ± 0.02
Soil III	34.70 ± 1.69 0.78 ± 0.04	33.86 ± 5.94 0.59 ± 0.05	35.20 ± 1.06 1.15 ± 0.04	34.47 ± 1.03 0.90 ± 0.08	36.45 ± 1.19 0.98 ± 0.03
Soil IV	35.66 ± 0.59 0.56 ± 0.03	37.36 ± 1.13 0.49 ± 0.08	34.54 ± 0.70 0.82 ± 0.07	36.53 ± 1.33 0.92 ± 0.05	38.15 ± 1.81 0.87 ± 0.04
Ni (mg·kg^−1^)					
Soil I	5.83 ± 0.11 2.28 ± 0.11	3.25 ± 0.06 2.01 ± 0.01	3.31 ± 0.09 2.01 ± 0.05	2.46 ± 0.10 1.54 ± 0.01	3.39 ± 0.03 1.85 ± 0.02
Soil II	4.62 ± 0.07 1.98 ± 0.07	2.65 ± 0.03 1.58 ± 0.03	3.68 ± 0.21 2.11 ± 0.04	3.48 ± 0.16 1.63 ± 0.02	3.50 ± 0.23 1.52 ± 0.11
Soil III	3.77 ± 0.03 1.74 ± 0.03	2.58 ± 0.12 1.24 ± 0.02	4.09 ± 0.03 1.58 ± 0.02	3.05 ± 0.05 1.58 ± 0.03	3.15 ± 0.06 1.85 ± 0.01
Soil IV	3.51 ± 0.12 2.02 ± 0.07	2.63 ± 0.56 1.22 ± 0.01	4.20 ± 0.02 2.03 ± 0.01	2.64 ± 0.24 1.01 ± 0.01	3.50 ± 0.22 1.12 ± 0.11

**Table 4:** Correlation matrix between physico-chemical properties of soil and plant-related parameters.

Variables	Biomass	Cr ^a^	Cr ^b^	Cr ^c^	Cr ^d^	pH	Cu ^c^	Cu ^d^	Zn ^c^	Zn ^d^	Ni ^c^	Ni ^d^
Biomass	**1**											
Cr ^a^	‒0.161	**1**										
Cr ^b^	0.180	**0.757**	**1**									
Cr ^c^	‒0.214	**0.821**	**0.759**	**1**								
Cr ^d^	‒0.245	**0.884**	**0.771**	**0.931**	**1**							
Ph	0.044	‒0.004	0.298	0.113	0.085	**1**						
Cu ^c^	‒0.077	**0.546**	**0.481**	**0.574**	**0.621**	‒0.012	**1**					
Cu ^d^	‒0.221	**0.519**	0.230	**0.564**	**0.673**	‒0.238	**0.620**	**1**				
Zn ^c^	0.084	0.248	0.151	0.093	0.150	0.089	‒0.119	0.161	**1**			
Zn ^d^	0.145	‒0.109	‒0.095	‒0.108	‒0.082	**0.501**	‒0.080	‒0.028	0.327	**1**		
Ni ^c^	‒0.247	‒0.199	‒0.263	‒0.166	‒0.194	‒0.261	0.173	0.097	0.139	‒0.337	**1**	
Ni ^d^	0.236	‒0.322	‒0.129	‒0.287	‒0.224	0.340	0.056	‒0.129	‒0.398	0.170	‒0.005	**1**

Values in bold are different from 0 with a significance level of alpha = 0.05. ^a^ metal concentrations in the above-ground plant material; ^b^ metal concentrations in the root; ^c^ total metal content in soil; ^d^ CaCl_2_-extractable metal concentration.

**Table 5:** Analysis of variance (ANOVA) results.

	Sum of Squares	df	Mean Square	F	Sig.
Biomass	2620.784	4	655.196	1.930	0.158
Cr ^a^	124.377	4	31.094	0.216	0.925
Cr ^b^	4519.891	4	1129.973	0.697	0.606
Cr ^c^	185.470	4	46.368	0.032	0.998
Cr ^d^	70.341	4	17.585	0.048	0.995
Ph	19.417	4	4.854	89.631	**0.000**
Cu ^c^	27.475	4	6.869	2.140	0.126
Cu ^d^	3.900	4	0.975	3.568	**0.031**
Zn ^c^	37.973	4	9.493	1.817	0.178
Zn ^d^	142.491	4	35.623	19.332	**0.000**
Ni ^c^	7.408	4	1.852	5.815	**0.005**
Ni ^d^	0.031	4	0.008	1.580	0.231

^a^ metal concentrations in above-ground plant material; ^b^ metal concentrations in roots; ^c^ total metal content in soil; ^d^ CaCl_2_-extractable metal concentration.
